# Force‐Triggered Non‐Volatile Multilevel Mechano‐Optical Memory System for Logic Computation and Image Recognition

**DOI:** 10.1002/advs.202413409

**Published:** 2025-02-17

**Authors:** Jiaxing Guo, Feng Guo, Hang Yang, Tianhong Zhou, Xiaona Du, Rui Gao, Haisheng Chen, Minghao Hu, Weiwei Liu, Yang Zhang, Dong Tu, Jianhua Hao

**Affiliations:** ^1^ Institute of Modern Optics and Tianjin Key Laboratory of Micro‐Scale Optical Information Science and Technology Nankai University Tianjin 300071 P. R. China; ^2^ Department of Applied Physics The Hong Kong Polytechnic University Hung Hom Hong Kong 999077 P. R. China; ^3^ Faculty of Materials Science and Chemistry China University of Geosciences 388 Lumo Road Wuhan 430074 P. R. China; ^4^ Institute of Photoelectric Thin Film Devices and Technology College of Electronic Information and Optical Engineering Nankai University Tianjin 300071 P. R. China; ^5^ Wuhan University Shenzhen Research Institute Shenzhen 518057 P. R. China

**Keywords:** boolean logic operations, in‐memory computing, mechanoluminescence, mechano‐optical memory, photostimulated luminescence

## Abstract

In the big data era, sensing multi‐modal information in memory is highly demanded for the sake of artificial intelligence applications to overcome the limitations of the von Neumann architecture. Different from traditional sensing methodologies, mechanoluminescence (ML) materials, which emit light in response to mechanical force without any external power supply, present intriguing prospects for technological developments. However, most of the ML materials only demonstrate instantaneous luminescence, severely hampering the exploitation of ML in sophisticated applications where non‐volatile control is indispensable. Herein, a non‐volatile, multilevel mechano‐optical memory system is proposed, based on a crafted combination of a self‐recoverable ML material, ZnS:Cu, and a photostimulated luminescence (PSL) phosphor Ca_0.25_Sr_0.75_S:Eu (CaSrS:Eu). By integrating ML with PSL effect, a robust six‐level non‐volatile memory is achieved, in which the multilevel memory states allow for computational capability without electrical interference. Specifically, the reliable multilevel and non‐volatile response enables Boolean logic operations. Furthermore, neuromorphic visual pattern pre‐processing is implemented, resulting in a substantial increase in recognition accuracy from 20% to 80%. These findings endow force‐responsive phosphors with memory capability, fully leveraging the capabilities of ML and offering a new strategy for developing mechano‐optical hardware and concepts for future intelligent applications.

## Graphical abstract

The integration of photostimulated luminescence with self‐recoverable mechanoluminescence materials endows the system with the capability to record and visually interpret mechanical information, concurrently exhibiting a six‐level non‐volatile memory architecture. This configuration showcases superior multi‐level and non‐volatile responses, which serve as the foundation for in‐memory computing. Moreover, the system demonstrates its efficacy in performing Boolean logic operations and neuromorphic pattern pre‐processing.

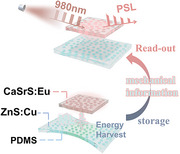

## 1 Introduction

Inspired by the human nervous system, sensing in memory represents a promising and energy‐efficient way to process external information for artificial intelligence applications. Mechanical signal sensing and processing are crucial for intelligent applications, such as human‐machine interfaces and robots, which are based on tactile information. To date, most measurements rely on electrical sensors made of piezoelectric, piezocapacitive, or piezoresistive materials, due to the requirement of precise and instantaneous mechanical sensing.^[^ ^1^, ^2^, ^3^, ^4^, ^5^, ^6^, ^7^ ^]^ However, the primary drawbacks of electrical sensors are that they require circuit access and an attendant power source. Besides, the separation between sensors and processing units in such conventional sensing systems leads to signal latency and significant energy consumption. Mechanoluminescence (ML) refers to the phenomenon of non‐thermal light emission triggered by mechanical stimuli such as grinding, rubbing, knocking, crushing, or pressing. As a direct mechano‐optical conversion effect, ML allows for the quantitative conversion of mechanical stimuli into light emission in a real‐time and in situ manner.^[^ ^8^, ^9^, ^10^, ^11^ ^]^ In recent years, through unremitting efforts, the brightness value of state‐of‐the‐art ML phosphors has reached 200–300 cd m^−2^ under a moderate mechanical force (such as finger touch), which is very close to the brightness of ordinary liquid crystal displays (LCDs) and mobile phone displays.^[^ ^12^, ^13^, ^14^ ^]^ Therefore, ML has paved the way for a wide range of promising applications, including self‐powered human‐machine interfaces, structural health monitoring and diagnosis, anti‐counterfeiting, bioimaging, and optogenetics.^[^ ^15^, ^16^, ^17^, ^18^, ^19^, ^20^, ^21^, ^22^, ^23^, ^24^, ^25^ ^]^ Nevertheless, most of the currently available ML materials exhibit instantaneous luminescence with a limited duration ranging from nanoseconds to microseconds. This instantaneous characteristic is beneficial for real‐time force sensing and monitoring technology. However, it is insufficient for the execution of more intricate experiments in which non‐volatile control over ML is indispensable.^[^ ^26^, ^27^, ^28^ ^]^

Implementing non‐volatile mechano‐optical memories can significantly enhance the performance in mechanical signal sensing and processing. A series of ML materials characterized by long lifetime or persistent ML have been proposed, which possess the potential to overcome the limitations of transient emitting behavior observed in conventional ML materials, extending the ML afterglow to tens of seconds.^[^ ^29^, ^30^ ^]^ Nevertheless, the persistent ML only provides a temporary and volatile memory functionality, with the non‐volatility remaining a challenging aspect. The trap‐controlled ML materials offer another potent solution to break this bottleneck. Mechanical force facilitates the liberation of electrons from the shallow traps into the conduction band. Some of the escaped electrons in the conduction band undergo recombination processes, resulting in instantaneous photon emission.^[^ ^31^ ^]^ Thus, for ML phosphors with suitable a trap‐depth distribution, some detrapped electrons would experience a redistribution process. Among these electrons, a fraction is recaptured by the relatively shallow traps, resulting in persistent ML. A small portion of the electrons, however, are almost irrevocably confined within the deep traps and cannot be thermally activated at ambient temperatures.^[^ ^32^, ^33^ ^]^ The electrons stored within the deep traps can be excited by near‐infrared (NIR) irradiation, releasing photons as photostimulated luminescence (PSL). The utilization of low‐energy NIR excitation enables a more controllable readout process and mitigates the measurement interference. Thus, the mechanically triggered PSL is an ideal candidate for mechano‐optical memories in potential information applications.^[^ ^34^ ^]^ Recent reports have demonstrated that, due to their broad trap‐depth distribution, deep‐trap ML materials enable non‐volatile storage of both the location and intensity of a pressure event.^[^ ^35^, ^36^ ^]^ These works pave the way for mechanical information storage and retrieval. Current developments in deep‐trap ML materials are addressing long‐standing challenges, including self‐powered visualization, non‐volatile storage, and optical readout of mechanical information. Due to the intrinsic trap distribution of these single‐phase deep‐trap materials such as BaSi_2_O_2_N_2_:Eu^2+[^ ^36^ ^]^ and SrSi_2_O_2_N_2_:Eu^2+^/Dy^3+^,^[^ ^35^ ^]^ after mechanically‐induced electron redistribution, only a small portion of electrons are stably stored in deep traps. The majority of electrons are trapped in relatively shallow traps, yielding immediate ML emission and short‐lived persistent luminescence. Moreover, all reported deep‐trap ML materials lack self‐reproducibility in the absence of light irradiation, which significantly hinders their practical applications. In the context of constructing ML‐based memory systems, a self‐charging ML capability is highly desirable, where neither pre‐ nor post‐irradiation steps are required. For the sake of convenience and practicability, optical readout is favored over the thermal excitation manner. However, achieving these criteria within a single‐phase ML compound presents a formidable challenge.

The functionality of setting optical responses to mechanical stimuli in a multistate, non‐volatile manner has not been reported yet. Herein, we demonstrate the feasibility of achieving self‐recoverable, force‐triggered, non‐volatile multilevel memory with optical accessibility through the meticulous integration of a self‐recoverable ML material, ZnS:Cu, and a PSL phosphor, Ca_0.25_Sr_0.75_S:Eu (CaSrS:Eu). PSL phosphors possess deep traps that can store electrons stably at ambient temperatures and release the stored electrons under light irradiation or thermal stimulus.^[^ ^37^, ^38^ ^]^ We show that the force‐triggered non‐volatile memory and multilevel operation can be achieved via coupling ML and PSL in a composite system. Our prototype devices can serve as non‐volatile multilevel mechano‐optical memories, programmable logic gates, and can perform neuromorphic pre‐processing for image recognition. These results provide a promising route to unleash the full potential of ML. The multilevel non‐volatile device sheds light on the implementation of new platforms for mechano‐optical signal processing.

## 2 Results and Discussion

### 2.1 Device Fabrication and Principles

**Figure 1:**
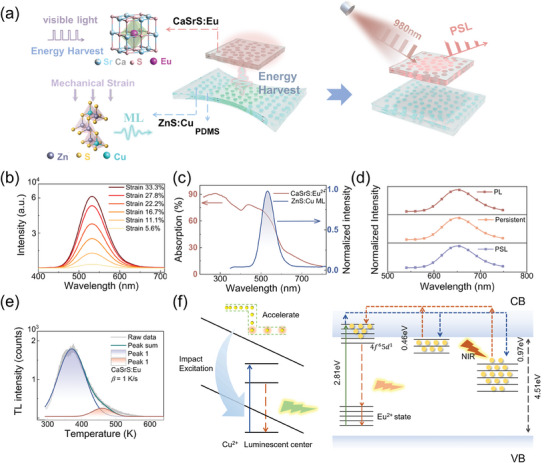
Luminescent characteristics of ML material ZnS:Cu, PSL material CaSrS:Eu and the working scheme of stress storage device. a) The schematic diagram of the mechano‐optical memory made of ZnS:Cu/CaSrS:Eu composites. b) ML spectra of ZnS:Cu/PDMS composite under different strains. The stretching‐releasing rate is fixed at 100 cpm. c) UV–vis absorption spectra of CaSrS:Eu and ML spectra of ZnS:Cu. The overlapping area between the two spectra is shaded. d) PL, persistent luminescence, and PSL spectra of CaSrS:Eu composite. e) TL glow curve of CaSrS:Eu at a heating rate of 1 K s^−1^ and the fitting curves. f) Schematic of physical mechanism of ZnS:Cu/CaSrS:Eu composites to realize storage and optical readout of mechanical information. The left subfigure of Figure 1f delineates the mechanism of the green emission from the ^4^T_1_–^6^A_1_ transition of Cu^2+^ ion, which is induced by triboelectricity effect. The green luminescence generated by ZnS:Cu can be reabsorbed by the adjacent CaSrS:Eu layer, and pump the electrons at the 4f levels to the 5d levels that overlap with the conduction band of CaSrS:Eu. These excited electrons will undergo redistribution to fulfill the storage and optical readout functions of the PSL layer, as illustrated in the right subfigure of Figure 1f.

The composite laminates are fabricated by integrating the ML material ZnS:Cu with the PSL phosphor CaSrS:Eu. **Figure** **1**a shows the schematic diagram of the mechano‐optical memory made of ZnS:Cu/CaSrS:Eu composites. In the composite system, the absorption spectrum of CaSrS:Eu (280–650 nm) overlaps with the principal wavelength range of visible light. The green emission centered at 525 nm from ZnS:Cu can be absorbed by the adjacent CaSrS:Eu layer, and this energy pumps the electrons to the conduction band of Eu^2+^. These excited electrons will undergo redistribution to achieve the non‐volatile multilevel memory and optical readout under the illumination of the 980 nm laser. Figures S1 and S2 (Supporting Information) show the X‐ray diffraction (XRD) patterns of ZnS:Cu and CaSrS:Eu powders, confirming that they are single‐phase materials with the dopants homogeneously distributed within the host matrices. To fabricate the ML composite film, ZnS:Cu particles and polydimethylsiloxane (PDMS) were mixed in a weight ratio of 6:4. The CaSrS:Eu phosphor was thoroughly mixed into the UV‐curable adhesive matrix at a weight ratio of 3:7 and coated onto the ZnS:Cu/PDMS laminate. Figure S3 (Supporting Information) depicts the schematic structure of the UV‐adhesive layer embedded with CaSrS:Eu particles, which is adhered to the ZnS:Cu/PDMS laminate (details in the Experimental Section). The back‐side of the ZnS:Cu/PDMS laminate was coated with silver paste to mitigate the scattering of ML, enhancing the reflection of ML into the CaSrS:Eu layer. Figure S4 (Supporting Information) shows the optical image of the resultant composite laminate. When the ZnS:Cu/PDMS composite was stretched or rubbed, intense ML emission could be observed. Figure 1b shows the ML emission spectra of the ZnS:Cu/PDMS composite obtained during stretching‐releasing periodic motions with different tensile strains. The stretching‐releasing rate was set at 100 cycles per minute (cpm). The green emission band centered at 525 nm arises from the impurity‐induced shallow donor state and the t_2_ state of Cu.^[^ ^39^ ^]^ The variation of the ML intensity almost maintains a linear relationship with the increasing strain (Figure S5, Supporting Information), which allows for the quantitative analysis of strain. Figure S6 (Supporting Information) confirms the stability and self‐reproducibility of ML from ZnS:Cu under continuous mechanical actuation (≈33.3% strain). CaSrS:Eu is regarded as a prominent PSL phosphor, characterized by its  superior electron‐trapping capability. Figure 1c shows the absorption spectra of CaSrS:Eu as well as the ML emission spectra of ZnS:Cu. The UV absorption spectrum observed below 300 nm arises from the band‐to‐band transition of CaSrS host. The absorption band observed in the blue/green region originates from the transition from the ground 4f^7^ state to the upper 4f^6^5d^1^ state of Eu^2+^ located at the octahedral site (O_h_) of the CaSrS lattice.^[^ ^40^ ^]^ As designed, the overlapping area in Figure 1c enables the PSL phosphor CaSrS:Eu to re‐absorb the ML photons emitted from the ZnS:Cu layer. Figure 1d shows the photoluminescence (PL) spectra under UV (365 nm) excitation, the persistent luminescence spectra after termination of the UV excitation, and the PSL spectra of the CaSrS:Eu composite. Notably, similar red emission bands centered at 649 nm unambiguously reveal that all of these emissions originate from the 4f^7^–4f^6^5d^1^ transition of Eu^2+^ ions. PSL spectra under 980 nm laser excitation from CaSrS:Eu were measured after the persistent luminescence completely disappears.

To delve deeper into the underlying mechanism of this phenomenon, we conducted thermoluminescence (TL) testing to determine the trap structure of CaSrS:Eu, which is essential for persistent luminescence and PSL. Figure 1e shows the TL glow curve of CaSrS:Eu at a heating rate (*β*) of 1 K s^−1^. The TL glow curve of CaSrS:Eu at 1.0 K s^−1^ heating rate can be decomposed into two Gaussian components centered at 371 and 460 K, respectively, suggesting that two trap depths exist. Utilizing Chen's equation,^[^ ^41^ ^]^ the corresponding trap depths were calculated to be 0.39 and 0.97 eV, respectively, which may be ascribed to the S interstitial (S_i_) and S vacancy (V_s_).^[^ ^42^, ^43^ ^]^ The shallow traps with a depth of 0.39 eV contribute to the persistent luminescence. Additionally, the deep traps with a depth of 0.97 eV exhibit sufficient stability to store the excitation energy at room temperature. Based on the aforementioned results and previous reports, the operational mechanism of the stress‐storage device can be depicted as illustrated in Figure 1f. After the ZnS:Cu/PDMS composite undergoes elongation and relaxation, the triboelectric effect gives rise to an amount of charge accumulated at the interface between the ZnS:Cu particle and the PDMS matrix. The resultant electric field is higher than the threshold value of the electric field (10^5 ^V cm^−1^) required to excite electroluminescence of ZnS:Cu.^[^ ^44^ ^]^ Thus, the observed green emission from the ^4^T_1_–^6^A_1_ transition of Cu^2+^ ion can be ascribed to the triboelectricity‐induced luminescence.^[^ ^45^ ^]^ The underlying mechanism of this process is clearly delineated in the left sub‐figure of Figure 1f. Due to the close proximity between the ZnS:Cu and CaSrS:Eu layer in the laminates, the green luminescence generated by ZnS:Cu can be effectively re‐absorbed by the adjacent CaSrS:Eu layer, as a result the electrons at the 4f levels of Eu^2+^ are pumped to the 5d levels, which overlap with the conduction band of CaSrS:Eu. These excited electrons will undergo redistribution to achieve the storage and computation functions of the PSL layer, as illustrated in the right subfigure of Figure 1f. Some electrons in the conduction band subsequently diffuse to the electron traps lying below the conduction‐band minimum of CaSrS. Shallowly trapped electrons easily escape back to the conduction band at room temperature and then are re‐captured by activators, resulting in a red afterglow emission. Deeply trapped electrons, however, are relatively stable and difficult to release at room temperature. Instead, they can be re‐activated by near‐infrared light irradiation or high‐temperature heating.^[^ ^46^ ^]^ Subsequently, the carriers captured by deep traps can be triggered to give rise to red PSL emission.

### 2.2 Device Performance Characterization

**Figure 2:**
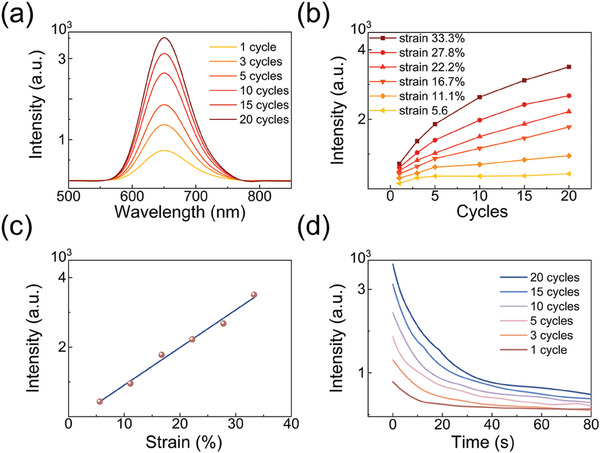
Persistent luminescence characteristics of ZnS:Cu/CaSrS:Eu composites under mechanical strain. a) The persistent luminescent spectra of the composite laminate under varying strain cycles are presented. With the tensile strain maintained at 33.3%, and the strain rate set at 100 cycles per minute (cpm). b) The persistent luminescent intensities exhibit a variation in relation to both the tensile strain and the number of stretching times. c) The persistent luminescent intensities are depicted as a function of the applied stretching strain. d) The persistent luminescence decay curves are plotted against the number of stretching times.

**Figure** **2** demonstrates the persistent luminescence characteristics of ZnS:Cu/CaSrS:Eu composite laminate actuated by mechanical force. The transient emitting behavior of ML requires instantaneous measuring equipment, which is conspicuously inconvenient in numerous application scenarios. Persistent luminescence can alleviate this stringent limitation, by introducing an additional time delay between the measurement point and the occurrence of the mechanical event.^[^ ^47^ ^]^ Figure 2a shows the persistent luminescent spectra of the composite laminate under repeated stretching. When the tensile strain was held constant at 33.3%, the red persistent luminescence intensifies progressively as the number of stretching cycles increases. Figure 2b unveils the relationship between the persistent luminescent intensity, the tensile strain, and the number of stretching cycles. As previously expounded, CaSrS:Eu, functioning as a persistent luminescent phosphor is capable of storing ML energy in shallow traps (0.46 eV). A larger tensile strain and a greater number of stretching cycles contribute to an increased quantity of electrons being trapped in the shallow traps, thereby leading to an enhancement in persistent luminescence. Figure 2c shows that the persistent luminescent intensities exhibit a linear increase with respect to the applied stretching strain. As shown in Figure 2d, the number of stretching times can also extend the decay curve of persistent luminescence due to a greater number of electrons being stored in the shallow traps. Figure S7 (Supporting Information) depicts the graphs of sample obtained at different time instants and their respective positions within the normalization decay curve subsequent to subjecting the sample to 10 stretching cycles (with a tensile strain of ≈33.3%). The persistent luminescence during the decay curve period can be readily observed by the naked eye or conventional photographic equipment. This property facilitates the visualization and quantitative assessment of mechanical activities without the need for immediate action.

**Figure 3:**
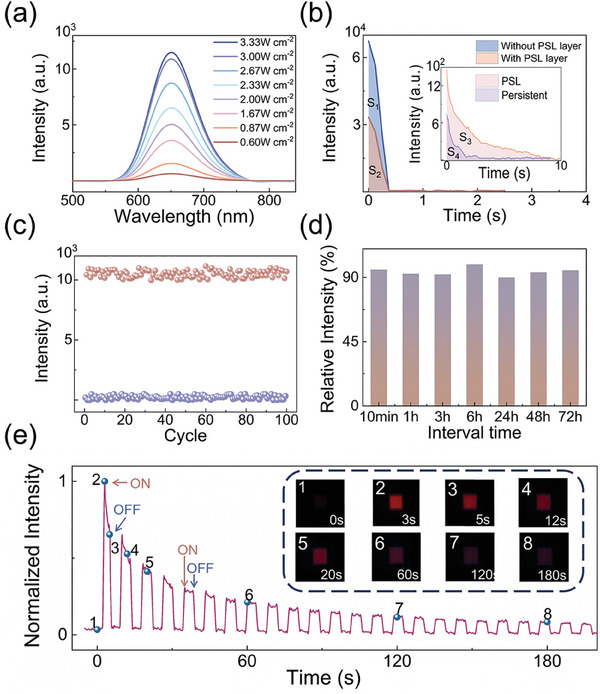
PSL characterizations of ZnS:Cu/CaSrS:Eu composites under mechanical strain. a) PSL spectra under different NIR 980 nm excitation power densities. Prior to measurement, the sample was adequately charged by undergoing 20 stretching times at a 33.3% strain. b) PSL efficiency of the composite system. The S_1_ and S_2_ areas represent the ML emissions from ZnS:Cu layer without and with CaSrS:Eu PSL layer during one stretching‐releasing cycle, respectively. The inset of this figure shows the persistent luminescence (area S_4_) and PSL (area S_3_) emitted from the CaSrS:Eu composite layer under one stretching‐releasing cycle. PSL efficiency can be calculated as S_3_/(S_1_‐S_2_) ^*^100% = 38%. c) PSL intensity variation upon alternating 980 nm light irradiation (3.33 W cm^−2^) and mechanical stimuli (33.3% strain for 20 times). Purple circles represent the PSL intensity after 980 nm light irradiation for 100 s, while the orange circles represent the PSL intensity after mechanical stimuli. d) Optical readout PSL intensities at different times after ceasing the mechanical stimuli. The PSL intensities maintain stable over 72 h when the composite laminates are preserved at room temperature and shielded from light. e) PSL response to periodical NIR light illumination. Insets show the photos of sample under 980 nm laser irradiation taken at corresponding moments.

Subsequently, our focus shifts to the PSL characteristics of the ZnS:Cu/CaSrS:Eu composite laminate, as shown in **Figure** **3**. For PSL measurement protocol, the sample was initially subjected to mechanical stretching for varying durations. Following the termination of persistent luminescence, The PSL spectra were recorded under continuous 980 nm laser irradiation. Figure S8 (Supporting Information) displays the PSL emission spectra of the composite laminate under various stretching cycles. During the measurement process, the stretching strain was maintained at 33.3%, and the power density of the exciting 980 nm laser was held constant at 3.33 W cm^−^^2^. Figure S9 (Supporting Information) depicts that the PSL intensity exhibit an increase with the stretching strain and the number of cycles. When subjected to mechanical strain, the doped Eu^2+^ activators are optically activated by ML emission. Electrons trapped in shallow levels are released as persistent luminescence. In contrast, electrons trapped in deep levels are relatively stable and can be excited by NIR light irradiation. Under conditions of larger strain and a greater number of stretching times, a larger quantity of electrons are trapped in the deep traps, thereby leading to an enhancement in PSL intensities. Figure S10 (Supporting Information) demonstrates that, in the absence of mechanical strain, no PSL emission can be observed from ZnS:Cu/CaSrS:Eu composite under 980 nm laser excitation (≈3.33 W cm^−2^). We further measured the PSL spectra of pure CaSrS:Eu composite film under mechanical strain, as presented in Figure S11 (Supporting Information). After 10 cycles of mechanical stretching, no luminescence can be detectable under 980 nm laser excitation. Consequently, the influence of triboelectricity can be conclusively excluded. Figure 3a shows the dependence of PSL intensity on the excitation power density. Prior to the measurement, the sample was adequately charged by undergoing 20 cycles of stretching at a 33.3% strain. The PSL intensity exhibits a low‐power density threshold of 0.60 W cm^−2^. Subsequently, it increases along with the increase of the pump laser power density and gradually reaches saturation at a power density of 3.33 W cm^−2^. A higher excitation power density pumps more trapped electrons into the conduction band of CaSrS:Eu. These electrons are subsequently captured by Eu^2^⁺ activators, ultimately resulting in the enhancement of PSL. The saturated PSL can be ascribed to the dynamic equilibrium between the electron‐releasing and electron‐filling processes at traps under a high pumping photon flux. Figure S12 (Supporting Information) shows the PSL decay curves of the mechanically charged composite laminate after different stretching‐releasing cycles. The stretching strain and excitation power density were set at 33.3% and 3.33 W cm^−2^, respectively. Under moderate excitation, the PSL emission can persist for over tens of seconds until the exhaustion of deeply trapped electrons. More importantly, a PSL signal can be detected after only one stretching‐releasing cycle, indicating that the ZnS:Cu/CaSrS:Eu composite possesses relatively high strain sensitivity and energy‐harvesting efficiency. We also estimate the PSL efficiency of the composite system. As shown in Figure 3b, the area marked as S_1_ represents the ML emission from ZnS:Cu layer without CaSrS:Eu PSL layer during one stretching‐releasing cycle. The area marked as S_2_ is the ML emission from ZnS:Cu covered with CaSrS:Eu composite layer. Since the back side of the ZnS:Cu/PDMS laminate was coated with silver paste, ML emission is reflected by the silver paste and can only be observable from the front side. Ignoring the ML scattering within the composite, the absorption fraction can be deduced from the difference between S_1_ and S_2_. The inset of Figure 3b shows the persistent luminescence (area S_4_) and PSL (area S_3_) emitted from the CaSrS:Eu composite layer during a single stretching‐releasing cycle. Thus, the PSL efficiency can be calculated as S_3_/(S_1_‐S_2_)^*^100% = 38%. The data retention capability of the sample was verified by monitoring the PSL intensities over time. Figure 3c presents the variation in PSL intensity of the sample when subjected to alternating 980 nm light irradiation and mechanical stimulus for 100 cycles. It is observed that the fluctuations in PSL emission following cyclic modulation remain stable, demonstrating the reversible PSL‐switching capability. As depicted in Figure 3d, the PSL intensities of the mechanically‐charged samples maintain relatively stable over 72 h when the composite laminates are stored at room temperature and shielded from light. The ZnS:Cu/CaSrS:Eu composite demonstrates excellent non‐volatile capability. In addition to the non‐volatility, the ZnS:Cu/CaSrS:Eu composite demonstrates a well‐behaved multilevel response. Figure 3e shows the PSL response of the charged CaSrS:Eu composite to periodic NIR light illumination. When the 980 nm light is turned on, bright PSL emission is observed, which gradually fades away. The decay time constant Λ is calculated to be 0.75 s.^[^ ^48^ ^]^ The PSL emission ceases instantaneously when the 980 nm light is turned off. Upon re‐activation of the light irradiation, the PSL emission reappears immediately, with its intensity matching that at the previous turn‐off moment. This suggests that the composite offers a stress‐triggered platform capable of exhibiting non‐volatile and multilevel optical responses.

### 2.3 Device Applications

The ZnS:Cu/CaSrS:Eu composite, serving as a pressure‐sensitive memory medium, can greatly enhance the capability for stress recording across a broader timescale. As a proof of concept, we designed and constructed two devices for stress‐recording applications. As presented in Figure S13 (Supporting Information), we utilized the CaSrS:Eu composite to write the letters “NK” onto a ZnS:Eu/PDMS plate. After stretching and releasing the composite film for 10 cycles, a clear instantaneous green ML emission was observed from the ZnS:Cu/PDMS plate, excluding the areas covered by the letter “NK”. Following the cessation of mechanical stimulation, the persistent luminescence emitted by CaSrS:Eu enables the delayed measurement of the stored information. After a waiting period of 300 s for the persistent luminescence to decay sufficiently, this combination leverages the PSL effect to realize an optical readout of the mechanical information on demand, without the need for any pre‐irradiation. Thus, three measurement modes: real‐time, delayed, and on‐demand measurements have been achieved in the fabricated stress sensor. Our composite system is far more stable and practical than the single‐phase deep‐trap materials that require light pre‐irradiation. We show that a rewritable non‐volatile photomemory can be constructed to convert information‐encrypted mechanical signals into visible patterns via photonic readout. For instance, a string of letters “Moving On” can be encoded with standard eight‐bit ASCII characters composed of different combinations of zeros and ones. As shown in Figure S14 (Supporting Information), the input binary codes “1” and “0” correspond to the presence or absence of mechanical knocking. The conversion of mechanical signals “Moving On” into a visible pattern was realized through NIR light irradiation. Another noteworthy aspect is that the proposed photomemory possesses a burn‐after‐reading feature. When the stored electrons are exhausted, this feature holds promise for applications in information encryption and optical anti‐counterfeiting. There is still considerable potential for enhancing the storage density of the prototype device by reducing the size of the memory unit.

**Figure 4:**
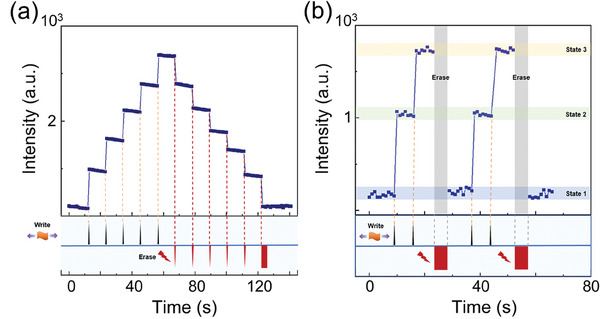
Multilevel operation of the mechano‐optical memory. a) Demonstration of six distinguishable non‐volatile states written by a series of mechanical stress pulses. The read operation is realized by 980 nm laser pulse (2.0 W cm^−2^, 100 µs duration). The erase steps are carried out using 980 nm laser pulses with a power density of 3.33 W cm^−2^. b) Reproducibility of the multilevel memory states. The reproducibility of multilevel memory states for cyclic write‐read‐erase operations shows a tendency to be stable.

We have gone a step further by demonstrating that a single device can realize multilevel storage operation. The device is initialized to state 1 when the composite is free from mechanical stimuli. By applying sequential strain pulses to the composite, we monitor each state by measuring the corresponding PSL intensities under 980 nm laser excitation. In order to keep the storage state stable, we use a 980 nm laser pulse (with a pulse width of 100 µs and a power density of 2.0 W cm^−2^) to optically read out the storage state. As shown in **Figure** **4**a, six distinct states can be mechanically programmed and clearly distinguished. Besides write and read operations, the storage information can be optically erased by illuminating the composite with a 980 nm laser at a higher power density of 3.33 W cm^−2^. Several intermediate levels can be freely accessible through a designed erase operation. After exhausting the trapped electrons, the memory state returns to the initialized state 1. To demonstrate the repeatable setting of multiple states, we repeat the two‐cycle write‐read‐erase operations, as shown in Figure 4b. This demonstrates the reliable reproducibility of the multilevel memory states. Figure S15 (Supporting Information) further examines the stability of the device's switching between different states during six cycles. These results confirm the repeatability and stability of setting multiple states in the mechano‐optical memory. Force‐triggered multilevel mechano‐optical memories allow for a greater storage density and provide more computational capability, which has not been achieved by any previous form of direct mechano‐optical memory.

**Figure 5:**
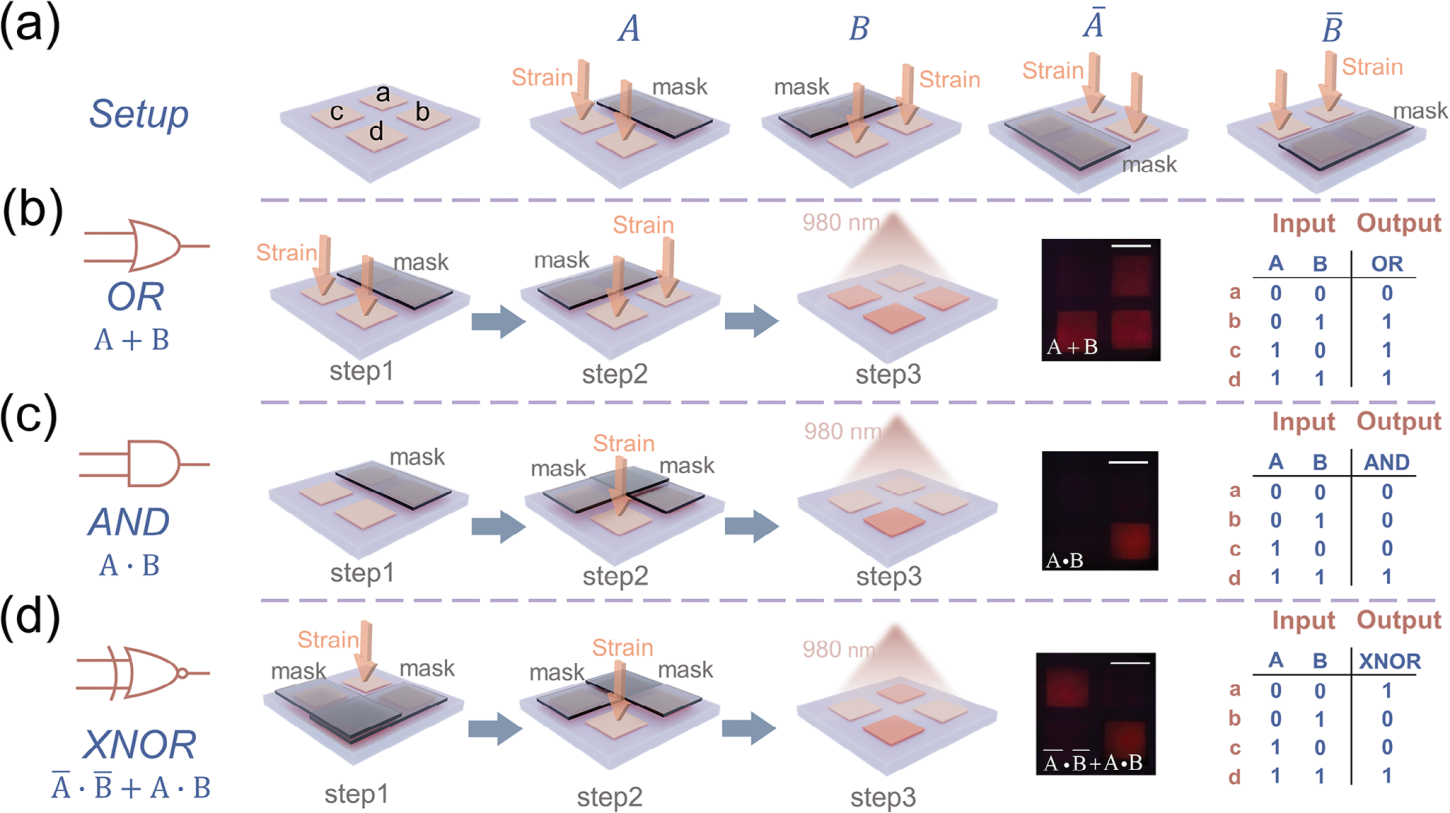
Working principle and demonstrations of logic AND, OR, and XNOR operation on 2 × 2 array device. a) The fundamental configuration of the 2 × 2 array device and the basic logic operation NOT. b), c) and d) are diagram and operating principle of logic OR, AND and XNOR operation, respectively. The corresponding truth table and the photograph of the experimental results are illustrated in the two right‐most images. The scale bar in the photograph is 3 mm.

Boolean logic operations form the foundation of computer programming, typically involving one or two inputs and a single output. Here, we utilize the ZnS:Cu/CaSrS:Eu composite as an example to demonstrate Boolean logic operations with mechanical inputs. To visually display the operation results, a 2 × 2 array device is employed for executing four‐bit binary Boolean logic operations. In our design, the application or non‐application of stress serves as the mechanical input. The logic out TRUE (1) and FALSE (0) are defined according to whether the device produces any PSL output. **Figure** **5**a illustrates the fundamental configuration of the 2 × 2 array device and the operational implementation of the basic function NOT. The four distinct binary arithmetic units are labeled as (a,b,c,d). The mask positioned at the top signifies the four‐bit binary input A (0011), while the mask to the left represents the four‐bit binary input B (0101). The NOT operation involves the displacement of the mask to the other two operation units. Figure 5b presents a schematic diagram of the basic logic operation OR. The device is subjected to two separate inputs of mechanical information under the mask of binary input A and input B, respectively. The four‐bit binary output (0111) is detected by a 980 nm light source. The photograph of the experimental results and the truth table are shown in the two images on the right‐hand side of Figure 5b, and the scale in the photograph is 3 mm. Regarding the basic logic operation AND, the device is subjected to inputs of mechanical information under the superimposed mask of binary input A and input B. The output result (0001) is read by means of a 980 nm light source. The schematic diagram, experimental result photograph, and truth table are respectively shown in Figure 5c. Through the appropriate combination of the three basic logic operations OR, AND, NOT, complex Boolean logic operations can be derived. XNOR is used here as a specific example to illustrate compound logic operations. The XNOR logical operation can be achieved through the combination of basic logical operations, with the specific expression being: F =$\bar{A}\;\cdot \bar{B}$+A∙B. The implementation of XNOR is depicted in Figure 5d. Initially, the device is subjected to inputs of mechanical stimulation under the superimposed mask of binary input $\bar{A}$ and input $\bar{B}$. Subsequently, the device undergoes a second set of mechanical stimulation inputs under the superimposed mask of binary input A and input B. The experimental results (1001) are visually presented in the right‐most Figure 5d, and the corresponding truth table is displayed in the adjacent figure. Other Boolean logic operations including NAND, NOR, and XOR have been experimentally verified, and the experimental results are shown in the Figure S16 (Supporting Information). According to the above results, the device demonstrated its capability to execute both force‐triggered binary basic logic operations and complex logical compositions, paving the way for addressing intricate mechanical challenges.

**Figure 6:**
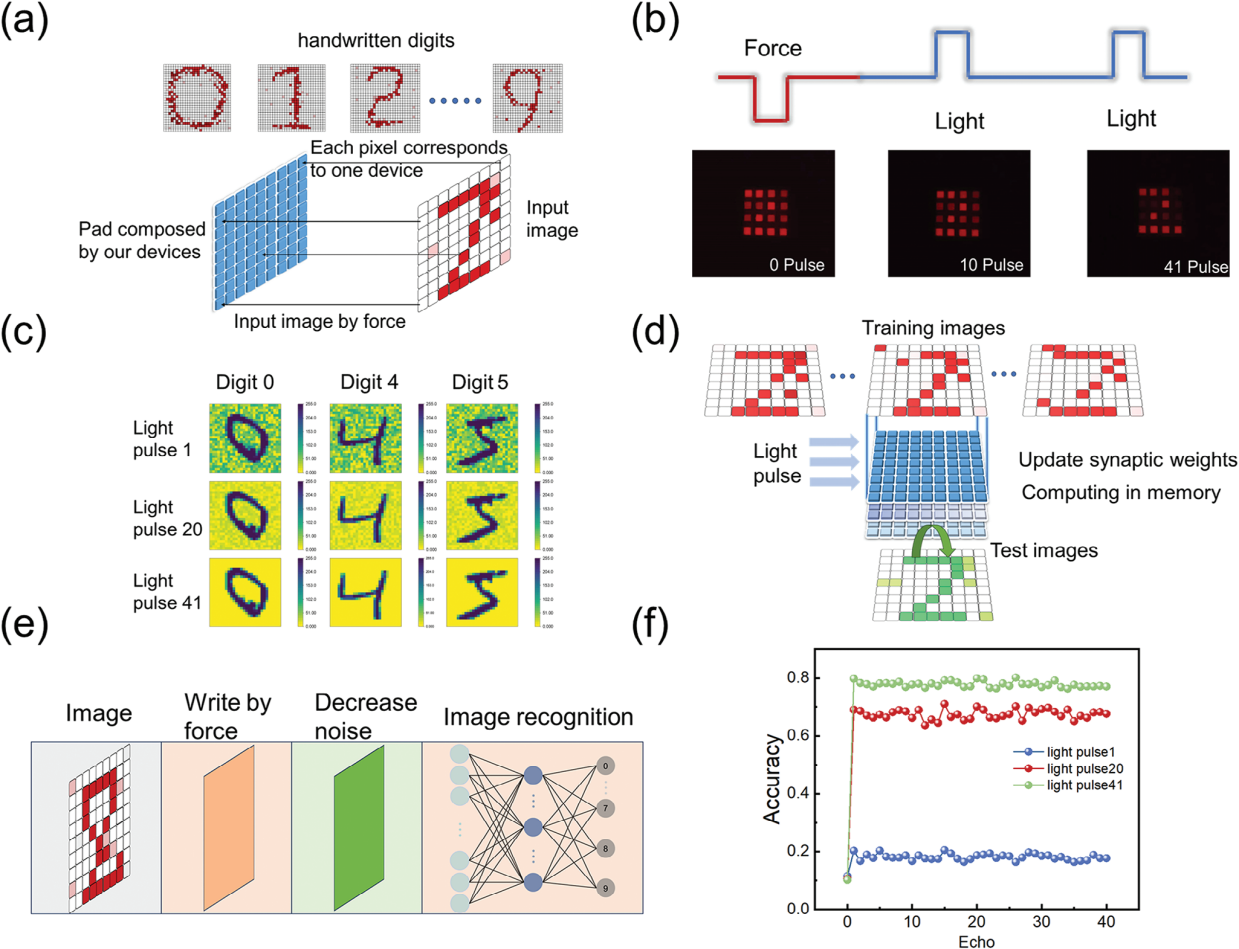
ZnS:Cu/CaSrS:Eu composites are utilized for sensing, processing, and recognizing handwritten digits. a) A device matrix is constructed to function as a graphic tablet, enabling the recording of a digit input via force. b) The digit on the graphic tablet can be read, and noise reduction can be achieved using 980 nm light, as shown in the real‐life photos. c) As the number of applied light pulses increases, the noise gradually disappears. d) A multilayer device matrix is used for image recognition by updating the synaptic weight through light modulation. e) The working principle of an artificial neural network based on the characteristics of device. f) The simulation results show the capability of device matrix for enhancing the image recognition accuracy.

Sensing and computing within memory represents a promising approach to surmount the bottleneck of contemporary machine vision grounded in the conventional von Neumann architecture. Unlike the prevailing methods that rely on optoelectronic devices to perceive mechanical patterns and process them with electrical signal, a mechano‐optical coupling approach is proposed by us for the first time. **Figure** **6**a depicts the working principle of a graphics tablet incorporating our devices. In this context, the MNIST handwritten digits are used as the images to be sensed. Each device within the graphic tablet corresponds to a pixel of the input image, due to the device's force‐sensing capability. Consequently, a handwritten digit can be recorded by our device matrix. Generally, the noise induced by the external environment and internal signal interference is inevitable. For improving the signal‐to‐noise ratio and sharpening the input image, the function of reducing the noise in the image is demonstrated, leveraging the effect of 980 nm light on the release of carriers within the material. As shown in Figure 6b, when force is applied to the graphics tablet, the digit “2” is recorded. Subsequently, a 980 nm laser pulse is imposed on the device matrix. In comparison to the pixels that have been written, the traps of noisy pixels capture a smaller quantity of carriers, leading to a faster depletion process. Consequently, the signal‐to‐noise ratio can be enhanced. Handwritten digits, consisting of 28×28 pixels arranged in accordance with the resolution of our device, are shown in Figure 6c. The maximum brightness of a pixel is 255 and the minimum is 0. After writing a number on the graphics tablet with a background noise level of 0.4 times the normal intensity, the digit is visualized by a light pulse designated as “light pulse 1,” where heavy noise can be observed. As the number of applied light pulses increases, the noise can be completely eliminated upon the application of the 41st light pulse. Another advantage of our device lies in its capacity to perform in‐memory computing using light signals rather than electrical signals, which effectively mitigates the signal delays and interferences. As shown in Figure 6d, upon feeding the training database into the device matrix, the synaptic weight is updated via modulating the light brightness. Subsequently, the test database is used to validate the image‐recognition accuracy. According to the abovementioned working principle, the workflow of sensing, processing, and recognizing the handwritten digits is depicted in Figure 6e. Initially, the digits are inscribed on the graphic tablet by the application of force. Then the noise is attenuated by repeated 980 nm light pulses. At last, the sharpened images are recognized through the device matrix through neuromorphic computing. Each device matrix can be considered as a neural‐network layer. In order to verify the capacity of our device for performing neuromorphic computing, a three‐layer artificial neural network is constructed based on the characteristics of the device. The input layer consists of 784 input neurons, while the hidden layer and the output layer are composed of 300 and 10 neurons, respectively. The synaptic weight is updated by light pulse in accordance with the backpropagation algorithm. The results in Figure 6f show that the handwritten digit recognition accuracy can be enhanced from ≈20% to 80% subsequent to the attenuation of noise in images. The mechano‐optical coupling ability of the device provides a new strategy for integrating sensing, processing, and in‐memory computing at the single‐device level for biomimetic multi‐sensory perception and next‐generation robotic intelligence.

## 3 Conclusion

ML materials inherently emit instantaneous luminescence, which imposes limitations on their applications in optical storage and processing. In this study, we present a prototype of a force‐triggered, non‐volatile mechano‐optical memory. This memory is founded upon a composite system that enables multi‐level storage and optical access. The system is constructed by integrating the ML phosphor ZnS:Cu with PSL phosphor CaSrS:Eu. Our proposed approach features self‐charging capability, distinguishing it from previously reported deep‐trap ML materials, such as BaSi_2_O_2_N_2_:Eu^2+^ and BaSi_2_O_2_N_2_:Eu^2+^/Dy^3+^, where pre‐ and post‐irradiation are essential. Furthermore, the energy storage and release processes of the PSL phosphor CaSrS:Eu exhibit enhanced stability, enabling repeatable NIR stimulated emission. The composite system exhibits high PSL efficiency and can response to a single mechanical stretching stimulation. Consequently, it holds promise for implementation in smart sensors and artificial intelligence platforms designed for human‐machine interfaces. Through precise control of the mechanical stimuli, non‐volatile, multilevel data can be inscribed into the memory with superior repeatability. Moreover, we demonstrate a six‐level long‐term stable memory state featuring optical readout and the capability for repeated write/erase cycling capabilities. This accomplishment paves the way for the development of artificial strain‐gated optical memory. In addition, our device showcases well‐behaved, multilevel, and non‐volatile response characteristics. As a result, it effectively accomplishes both neuromorphic visual pattern pre‐processing and intricate logic operations.

Potentially, the utilization of force as an alternative trigger presents a more straightforward, practical, and one‐step solution for scenarios such as biomimetic devices, in comparison to those relying on electricity or heat. As an information‐processing and storage strategy, unconventional mechanical computing can augment traditional electronic computing by incorporating novel mechanical systems. In our devices, ML or PSL (represented by specific physical parameters) is abstracted into binary numbers to sense, interact with, and process mechanical information in the environment. This strategy exhibits the capacity to interact with and adapt dynamically to environments that are beyond the reach of conventional electronic computing methods. Specifically, the self‐reproducibility of ML from ZnS:Cu endows the device with the autonomy to operate without reliance on an external power source. Its operation is manifested through optical signal readout, thereby eliminating the need for traditional circuitry and ensuring reliable performance under arduous conditions such as underwater environments, intense electromagnetic fields, and aerospace settings. These findings could contribute to the development of multi‐modal mechano‐optical hardware and concepts for information computing and artificial intelligence.

## 4 Experimental Section

### Phosphor Synthesis and Device Fabrication

ML phosphor ZnS:Cu was procured from Global Tungsten & Powders Co. PSL phosphor Ca_0.25_Sr_0.75_S:Eu (CaSrS:Eu) was synthesized by the traditional high‐temperature solid‐state method. The raw materials CaS (99.99%), SrS (99.99%), and EuCl_3_·6H_2_O (99.9%) were acquired and utilized without further treatment. Stoichiometric ratio of raw materials was weighed, blended, and meticulously ground for 30 min in an agate mortar. Subsequently, the homogeneous mixture was transferred into alumina crucibles and sintered in a corundum tube furnace at 1000 °C for 4 h under a N_2_ atmosphere. The ML phosphor composite was fabricated by homogeneously incorporating ZnS:Cu powders into the PDMS matrix at a weight ratio of 6:4. PDMS was formulated from its base and crosslink at a weight ratio of 10:1. The composite was then placed within a vacuum chamber for 30 min to eliminate the air bubbles. Subsequently, the ZnS:Cu/PDMS composite was cured at 80 °C for 2 h, followed by natural cooling. CaSrS:Eu phosphor was thoroughly mixed into the UV‐curable adhesive (Norland NOA 61) matrix at a weight ratio of 3:7 and spin‐coated on the ZnS:Cu/PDMS plate. Subsequently, the sample was irradiated with a UV lamp (365 nm) for 10 min to ensure complete curing.

### Material Characterizations and Photoresponse Measurements

An X‐ray diffractometer (Bruker D8 Discover: λ = 1.5406 Å, Cu Kα1 radiation) was employed to explore the crystal structure of the phosphors. Scanning electron microscopy (SEM) analysis was conducted using a Zeiss Sigma 500 field emission SEM system. The PL, ML, persistent luminescence, and PSL spectra were measured by means of a PyLoN CCD camera coupled with SpectraPro 300i spectrophotometer. The PL spectra were recorded under 365 nm laser excitation. During the ML measurement, the stretching‐releasing and knocking operations were achieved using a custom‐made mechanical drive system, as shown in Figure S17 (Supporting Information). A manipulator electric clamp MCE‐3G‐02‐050 was utilized to supply periodic strain. The PSL spectra were measured using 980 nm laser excitation. The laser power was measured using laser power and an energy meter (FieldMaxII‐TOP, Coherent). TL was measured by a fluorescence spectrometer (FLS1000, Edinburgh Instruments) with a versatile heating system (Linkam THMS600). The samples were irradiated with UV light (365 nm) for 1 min and then allowed to left for 3 min. Subsequently, the TL spectra were recorded at the optimal emission peaks. Luminescent decay curves were recorded by a photomultiplier (PMT) ((H11902‐04, Hamamatsu) coupled with Tektronix DPO3034 oscilloscope. The luminescence photos were captured using a mirrorless camera (Sony NEX‐5N) equipped with a Sigma (56 mm 1.4) lens.

## Conflict of Interest

The authors declare no conflict of interest.

## Supporting information



Supporting Information

## Data Availability

All data supporting the results of this study are available in the manuscript or the supplementary information. Additional data are available from the corresponding author upon request.
