# Supplementary material for: Force‐Triggered Non‐Volatile Multilevel Mechano‐Optical Memory System for Logic Computation and Image Recognition
Source: Adv Sci (Weinh). 2025 Feb 17;12(14):2413409. doi: 10.1002/advs.202413409 (PMC11984892; doi:10.1002/advs.202413409)
Supplement: Supplementary file 1 — Supporting Information [file ADVS-12-2413409-s001.docx]

Supporting Information

**Force-triggered non-volatile multilevel mechano-optical memory system for logic computation and image recognition**

Jiaxing Guo*^┼^*, Feng Guo*^┼^, Hang Yang, Tianhong Zhou, Xiaona Du, Rui Gao, Haisheng Chen, Yiyang Wen, Minghao Hu, Weiwei Liu, Yang Zhang, * Dong Tu *& Jianhua Hao **

J. Guo, T. Zhou, H. Chen, Y. Wen, M. Hu, W. Liu, Y. Zhang

Institute of Modern Optics and Tianjin Key Laboratory of Micro-Scale Optical Information Science and Technology, Nankai University, Tianjin 300071, P. R. China

F. Guo, J. Hao

Department of Applied Physics, The Hong Kong Polytechnic University, Hung Hom, Hong Kong, P. R. China

H. Yang, R. Gao, D. Tu

Faculty of Materials Science and Chemistry, China University of Geosciences, 388 Lumo Road, Wuhan 430074, P. R. China

X. Du

Institute of Photoelectric Thin Film Devices and Technology, College of Electronic Information and Optical Engineering, Nankai University, Tianjin 300071, China

D. Tu

Wuhan University Shenzhen Research Institute, Shenzhen, 518057, China

E-mail: *[yangzhang@nankai.edu.cn](mailto:yangzhang@nankai.edu.cn) ,*[tudong@cug.edu.cn](mailto:tudong@cug.edu.cn) and*[jh.hao@polyu.edu.hk](mailto:jh.hao@polyu.edu.hk)





**Figure S1**. X-ray diffraction (XRD) pattern of ZnS:Cu powders.





**Figure S2**. XRD pattern of CaSrS:Eu powders.


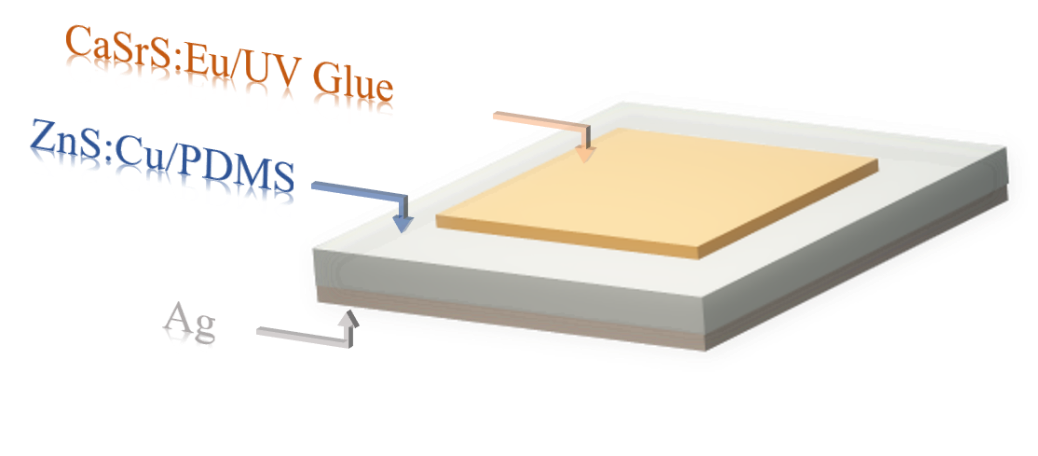


**Figure S3**. Schematic structure of the CaSrS:Eu /UV glue layer adhered to ZnS:Cu/PDMS laminate and Silver paste.


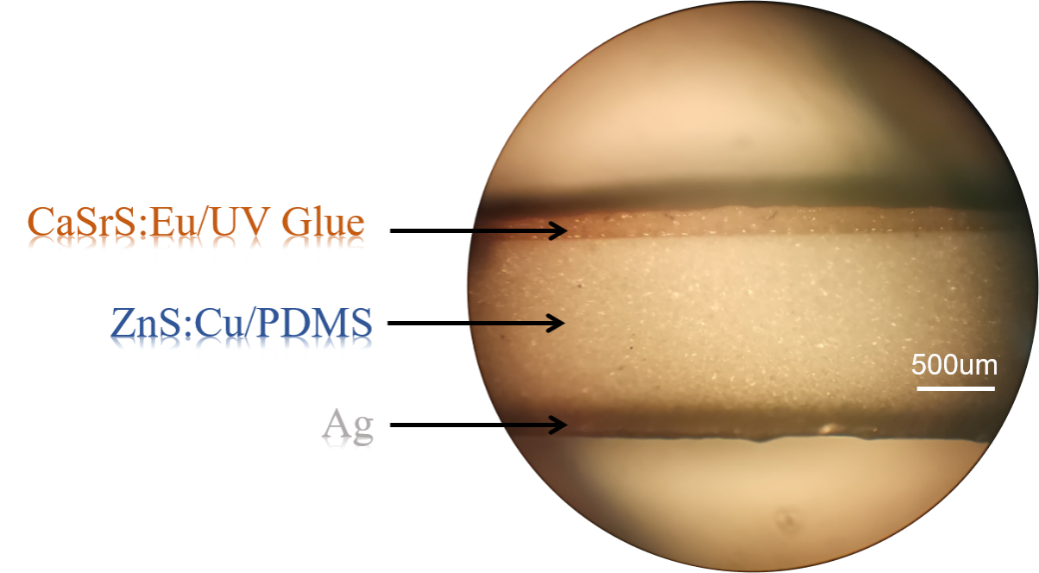


**Figure S4**. The optical microscope image of the resultant composite.





**Figure S5**. The variation of the ML intensity of ZnS:Cu with the increasing strain.





**Figure S6**. Stability and self-reproducibility of ML from ZnS:Cu under continuous mechanical strain.


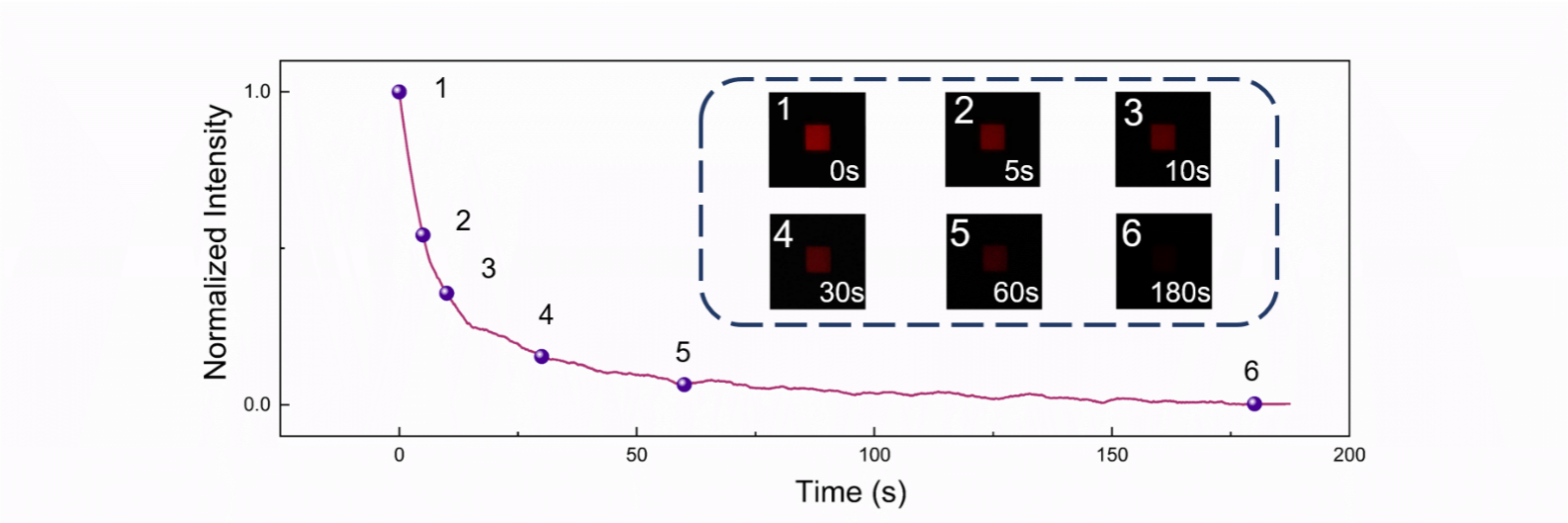


**Figure S7**. Persistent light emitting during the decay curve period. Insets show the captured pictures at different moments after ceasing mechanical stimuli.





**Figure S8**. The PSL emission spectra of the composite laminate under 33.3% strain with different stretching cycles.





**Figure S9**. PSL intensities versus the tension strain and stretching times. The readout NIR 980 nm excitation power density was fixed at 3.33 W/cm^2^.

**

**

**Figure S10**. No PSL emission can be observed in ZnS:Cu/CaSrS:Eu composite under 980 nm laser excitation (~3.33 W/cm^2^) when no mechanical strain is applied to the composite.





**Figure S11**. The ML spectrum from pure CaSrS:Eu composite layer under repeated mechanical strain. In the controlled experiment, no light emission can be observed in pure CaSrS:Eu composite layer.





**Figure S12**. PSL decay curve of the mechanically charged composite laminate after different stretching-releasing cycles. The stretching strain and excitation power density were fixed at 33.3% and 3.33 W/cm^2^, respectively.


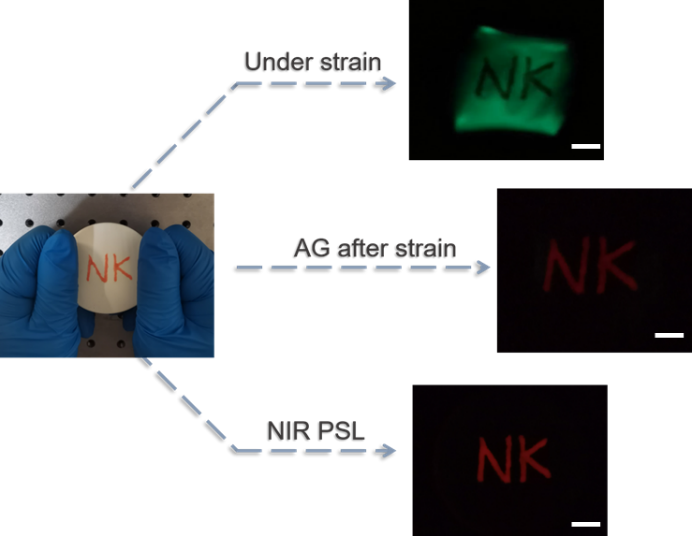


**Figure S13**. Mechanics storage and visualized reading activities with three measurement modes. Instantaneous ML for real-time measurement; Persistent luminescence for delay time measurement; PSL for on-demand measurement.


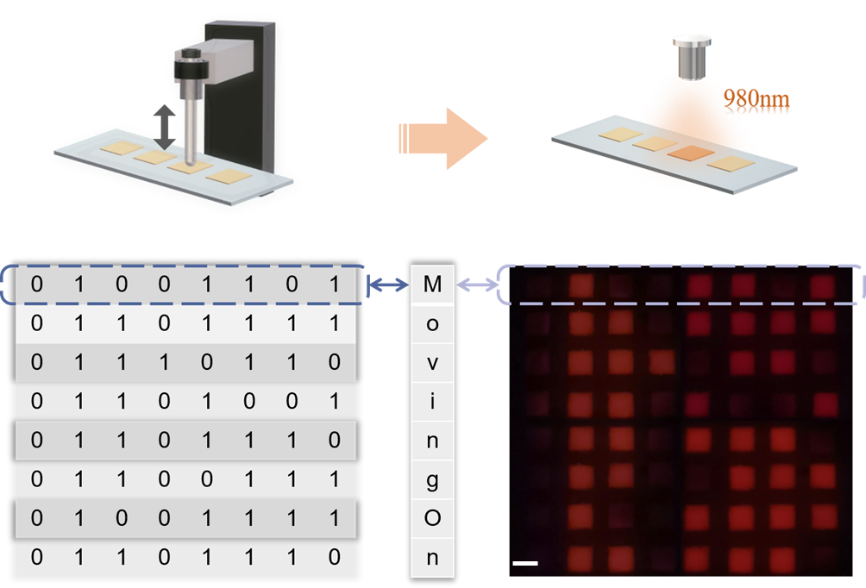


**Figure S14**. Digital encoding based on the mechano-optical conversion at ZnS:Cu/CaSrS:Eu composite laminate platform. Binary information processing of converting mechanical inputs which carry a string of letters “ Moving On” into optical signals. Scale bar: 5 mm.


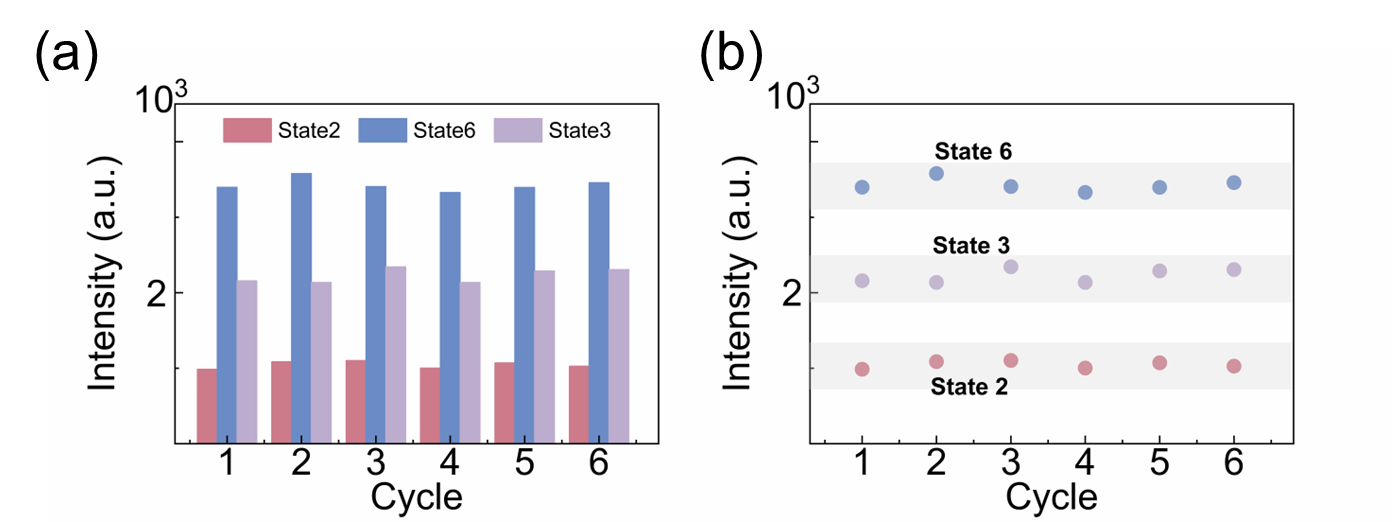


**Figure S15**. a) During six cycles, the device's response was switched from State 2 to State 6 and then readjusted back to State 3 using 980 nm laser pulses. b) The intensities of different states fluctuate within narrow ranges and can be clearly distinguished from each other.


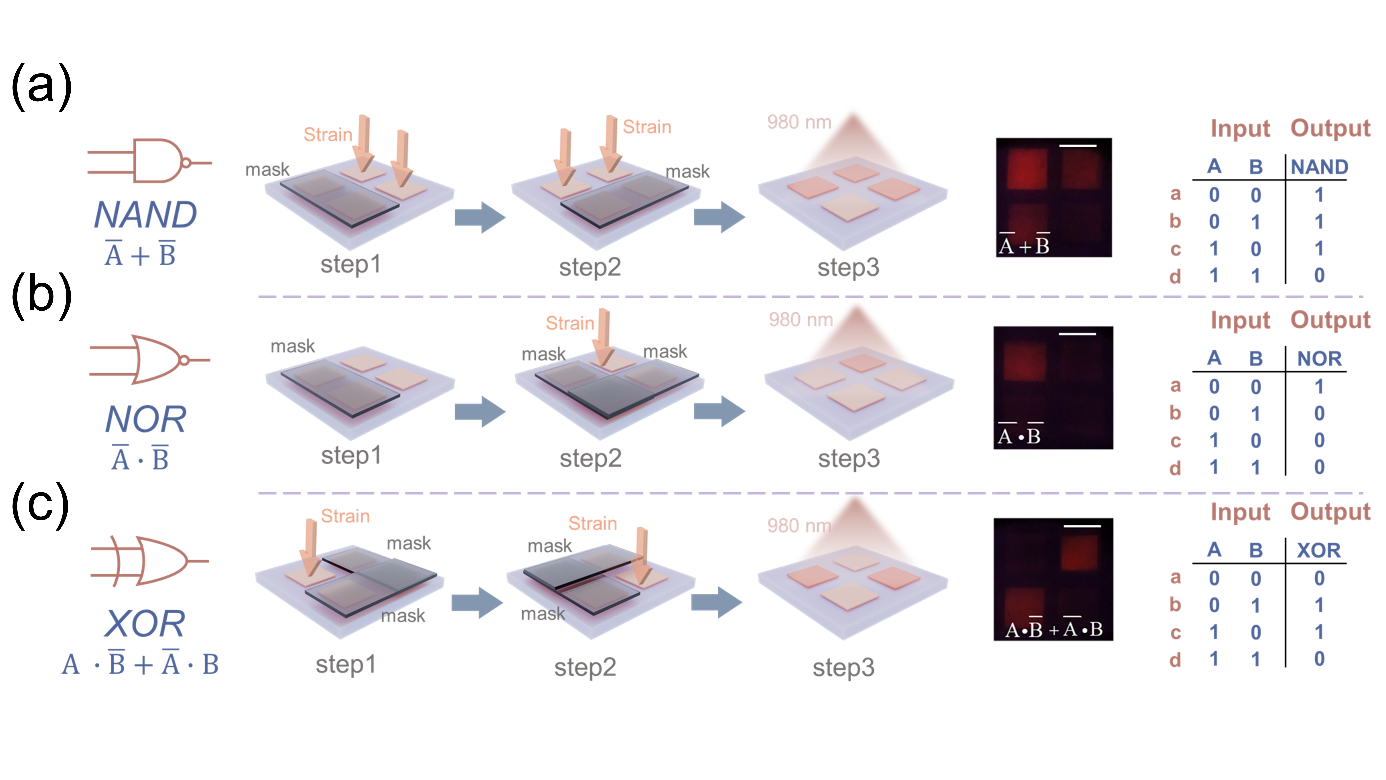


**Figure S16**. The diagram and operating principle of another 3 Boolean logic operations: a) NAND, b) NOR and c) XOR. The corresponding truth table and the photograph of the experimental results are shown in the two rightmost images. The scale bar in the photograph is 3 mm.


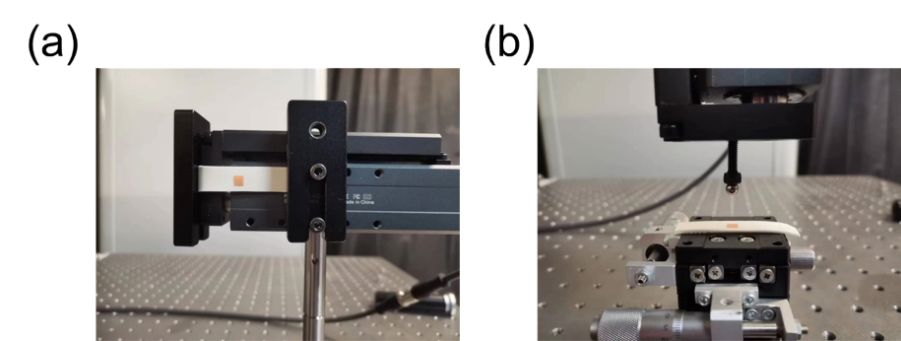


**Figure S17**. The graph of homemade mechanical stretching-releasing a) and knocking b) system.
